# The thromboprotective effect of traditional Chinese medicine Tongji 2 granules is dependent on anti-inflammatory activity by suppression of NF-κB pathways

**DOI:** 10.1371/journal.pone.0241607

**Published:** 2020-11-12

**Authors:** Lin Zhou, Stephanie Lapping, Xudong Liao, Yuan Lu, Guangjin Zhou, Keiichiro Matoba, Neelakantan Vasudevan, Lemin Wang, Lalitha Nayak

**Affiliations:** 1 Department of Cardiology, Tongji Hospital of Tongji University, Shanghai, China; 2 Case Cardiovascular Research Institute, Case Western Reserve University School of Medicine, University Hospitals Cleveland Medical Center, Cleveland, Ohio, United States of America; 3 Division of Diabetes, Endocrinology, and Metabolism, Department of Internal Medicine, The Jikei University School of Medicine, Tokyo, Japan; 4 Division of Hematology and Oncology, Case Western Reserve University School of Medicine, University Hospitals Cleveland Medical Center, Cleveland, Ohio, United States of America; Fiji national University School of Medicine, FIJI

## Abstract

Inflammation is a vital physiological response of the immune system meant to protect against the invasion of pathogens. However, accumulating evidence describes an intimate link between inflammation and thrombosis and cellular elements of the immune system of the immune system such as neutrophils and monocytes/macrophages are emerging as key players in the generation of a prothrombotic milieu suggesting that anti-inflammatory therapy may have a role in the management of thrombosis that is driven by inflammation. Tongji 2 (TJ2) is a traditional Chinese medication manufactured as granules by Tongji hospital of Tongji University (Shanghai, China) with known anti-inflammatory properties. In this study, we examine the effects of TJ2 on inflammation and thrombosis. Our study shows that TJ2 modulates NF-κB activation and thus generates a prominent anti-inflammatory effect. Further, we use mouse models of thrombosis to demonstrate that TJ2 has a beneficial effect in both arterial and venous thrombosis that occurs in the absence of alterations in platelet activation or coagulation.

## Introduction

Inflammation is a physiological response of the immune system that is primarily a means to prevent invasion of pathogens. This process involves activation and recruitment of cells of both the innate and the adaptive immune system that include monocytes/macrophages, neutrophils and lymphocytes. The activated immune cells, such as macrophages release numerous pro-inflammatory mediators and cytokines e.g. tumor necrosis factor-α (TNF- α), IL-1β and IL-6 [1, 2]. While these pro-inflammatory cytokines are essential to host defense, unbridled activation of the immune system can lead to unnecessary tissue injury and is now understood as the critical mediator of inflammatory disorders. NF-κB, the master regulator of inflammation, plays a pivotal role in the expression of inflammatory genes [3]. Not surprisingly, NF-κB is crucially involved in the pathogenesis of several chronic inflammatory disorders including autoimmune diseases, atherosclerosis, and even cancers [4, 5]. Hence, treatments aimed at inhibiting signaling pathways that involve NF-κB expression or activity may be a feasible approach in the treatment of certain inflammatory disorders.

Inflammation favors thrombosis, and accumulating evidence describes how inflammation generates a potent prothrombotic milieu [6, 7]. Immune and endothelial cells demonstrate increased expression of the potent pro-coagulant tissue factor (TF) in response to inflammatory stimuli, leading to increased thrombosis [8]. Thus, molecular mechanisms that link inflammation, cellular activation and thrombosis support the emerging theory of a thromboinflammatory process [9]. Consistently, venous thrombosis is noted with increased frequency in patients with chronic inflammatory disorders such as rheumatoid arthritis, highlighting the critical role of inflammation in these events as well [10]. Importantly, the critical role of inflammation in thrombosis is highlighted by the CANTOS trial where modulation of IL-1β signaling was associated with a significant decrease in recurrent cardiovascular events [11]. Cumulatively, these studies demonstrate the importance of inflammation in thrombosis and suggest that anti-inflammatory therapies may be an alternative approach to modulating thrombosis risk.

Traditional Chinese medicine views disease as a systemic disorder and treatment is usually geared towards modulating or restoring the immune response. Tongji 2 (TJ2) is a traditional Chinese medication manufactured as granules by Tongji hospital of Tongji University (Shanghai, China) [12]. This herbal preparation is comprised of pseudo-ginseng, astragalus mongholicus, and honeysuckle. Additionally, this preparation contains roots of Panax pseudo-ginseng Wall, also known as Sanchi, an herbal drug widely used in traditional Chinese medicine for the treatment of cardiovascular diseases. Animal studies show that this herb may increase coronary blood flow and reduce myocardial oxygen consumption [13]. The dried root of Astragalus membranaceus (Huangqi) is frequently used as an immunomodulating agent [14] in mixed herbal decoctions to treat common cold, diarrhea, fatigue and anorexia, and is also prescribed to patients with cardiac diseases [15]. Finally, Honeysuckle has been shown to possess anti-inflammatory properties [16].

Our study aimed to examine the effect of this herbal medication on inflammation, as well as arterial and venous thrombosis. The goals of this study were to explore (a) an in vitro anti-inflammatory effect of TJ2; (b) to examine if doses higher than that presently prescribed in humans has any hematological toxicity that could affect thrombosis assay or thrombosis risk; (c) the effect of TJ2 on thrombosis. Our results show that TJ2 modulates NF-κB activation and thus generates a prominent anti-inflammatory effect. Further, we show that TJ2 has a thrombo-protective effect that occurs in the absence of alterations in platelet activation or coagulation. The results of these studies have the potential to form the basis for a clinical trial that explores higher doses of the herbal preparation and toxicity studies, which so far has not been conducted.

## Materials and methods

### Cell culture and reagents

RAW 264.7 macrophages (American Type Culture Collection (ATCC), Rockville, MD, USA) were cultured in Dulbecco’s modified Eagle’s medium (DMEM) supplemented with 10% fetal bovine serum (FBS), 100U/ml penicillin, 100U/mL streptomycin and 2mM glutamine (Gibco-BRL, Grand Island, NY, USA) in a humidified atmosphere of 5% CO2 and 95% air at 37 °C. Cells were treated with lipopolysaccharide (LPS, Sigma-Aldrich) at indicated concentrations to induce an inflammatory response. Human umbilical vein endothelial cells (HUVECs) and human aortic endothelial cells (HAECs) (American Type Culture Collection (ATCC) (Rockville, MD, USA) were cultured in EGM-2 (Gibco-BRL, Grand Island, NY, USA) per manufacturer instructions. An inflammatory response was induced in the endothelial cell with interleukin -1 (IL-1, Sigma-Aldrich Chemical Co.). Human α-thrombin (3000 U/mg) was purchased from Haematologic Technologies. Rat antibodies to murine P-selectin (Wug.E9), the activated epitope of murine α_2b_β_3_ (JON/A) were purchased from Emfret Analytics. All other chemicals were purchased from Sigma-Aldrich unless otherwise indicated.

### Animals

Animals. Male C57BL/6J mice, aged 7–8 wk, were purchased from Charles River Laboratories (Wilmington, Massachusetts). Mice were housed in a temperature- and humidity-controlled specific pathogen–free facility with a 12-hour-light/dark cycle and ad libitum access to water and standard laboratory rodent chow. Mice were randomly assigned to two study groups. In mice receiving TJ2 treatment, the TJ2 group of mice underwent daily gavage with TJ2 (104mg/mouse/day) for two weeks. Vehicle group received equal volume of PBS. All animal studies were performed in mice 8–12 weeks of age. All in vivo studies were conducted within 24 after the last dose of TJ2. To assess the number of mice needed we extrapolated from our previous thrombosis studies performed in the laboratory. We are able to detect a significant difference in these mouse models (both arterial and venous thrombosis models) using 8–10 mice per group to achieve an α = 0.05 and β = 0.80.

The dose of TJ2 administered to mice was derived from the dose used in humans (10gm/70kg body weight) and calculated for mouse based on a previously published manuscript by Xiong [17]. The final mathematical conversion dose from human to mouse based on this study is 1:9/kg body weight. To elaborate, for every one milligram/kg body weight of the drug received by humans, the murine dose would need to be nine milligram/kg body weight. The traditional use of TJ2 in humans is at a dose of 10 gm per day. Thus, based on a body weight of 70 kg in humans, the final dose for a mouse that weighs 20gm is 25.7mg/day (rounded to 26mg/day). Thus, cohorts of mice (5 in each group), age 8 weeks, were treated by gavage daily at 26mg/day dose, two times this dose (52mg/day) and 4 times this dose (104mg/day) (solution was prepared as described below). Mice did not display any difference in body weight, overall appearance at the end of two weeks between all three doses. Complete blood counts were also checked and were no different in all three cohorts of mice. Hence, the highest dose (104gm/day) was used to treat mice that were tested for thrombosis assays as well as coagulation and platelet activation assays.

All animal experimentation was approved by the Case Western Reserve University Institutional Animal Care and Use Committee (Protocol number: 2018–0054). All animals were euthanized at the completion of the experiment with an overdose of anesthesia (isoflurane inhalation) followed by cervical dislocation.

### Preparation of TJ2 for cell culture and animal studies

TJ2 (Lot number 1305384) was provided by Tongji hospital of Tongji University (Shanghai, China). The active ingredients of TJ2 (Lot number 1305384) as determined by LC-MS/MS are as follows: chlorogenic acid 2373.84 ± 18.86 μg/g; notoginsenoside R_1_ 497.76 ± 12.64 μg/g; ginsenoside Rg_1_ 2678.84 ± 44.21 μg/g; ginsenoside Rb1 1657.46 ± 19.12 μg/g; astragaloside IV 48.75 ± 2.19 μg/g [12]. No assessments were made for heavy metals, pesticides or other toxic substances. To make a solution for cell culture, 2 g of TJ2 granules was added to 40 ml DMEM and mixed well. Mixture was then centrifuged (×3500rpm) for 5 minutes, supernatant transferred to a new tube and filtered with a 0.22 μm filter to obtain a 50 mg/ml stock solution. This was stored at -20°C and used for cell culture assays. For animal studies, TJ2 was added to PBS and the same procedure of preparation followed.

### MTS assay

Cell viability was measured based on the formation of formazan that is metabolized from colorless 3-(4,5-dimethylthiazol-2-yl)-5-(3-carboxymethoxyphenyl)-2-(4-sulfophenyl)-2H-tetrazolium,inner salt; (MTS; Promega Chemical Co.) Briefly, RAW 264.7 cells (5x10^5^ cells/ml) were seeded in a 96-well plate and treated with varying concentrations of TJ2 for three days. At the end of the treatment, cells were incubated with 20ul/well of combined MTS/PMS solution for 2 hours and absorbance at 490nm was recorded using an ELISA plate reader. Amount of absorbance is proportional to the number of viable cells. All treatment analyses were performed in triplicate.

### RNA extraction and qPCR

Total RNA was extracted from tissue samples using TRIzol reagent (Life Technologies, 15596–026) and from RAW 264.7 cells, HUVECs and HAECs using High Pure RNA Isolation Kit (Roche Diagnostics Corporation, USA) after the indicated treatments. Total of 2ug of total RNA was reverse transcribed using the iScript Reverse Transcription Kit (Bio-Rad, 170–8841). Real-Time PCR was performed using Universal SYBR Green PCR Master Mix on Applied Biosystems Step One Real-Time PCR System (Applied Biosystems) using gene specific primers. Relative expression was calculated using the ΔΔCt method with normalization to GAPDH or 18SRNA. The primers used for PCR are provided in S1–S3 Figs and S1 Raw Images.

### Protein extraction and western blot analysis

Nuclear and cytosolic proteins were prepared using NE-PER nuclear and cytoplasmic extraction reagents (thermo scientific, USA) according to the manufacturer’s instructions. The protein concentration of the cell lysate was determined using Pierce TM BCA protein assay kit (thermo scientific, USA). The Nuclear/ cytoplasmic portion of the cell lysate were separated by 10% PAGE gel and blotted with indicated antibodies.

### Blood cell count

Blood samples were collected in 3.8% sodium citrate. Complete blood count was measured using an automated analyzer (Sysmex XE5000, Kobe, Japan).

### Carotid artery thrombosis

Carotid artery thrombosis was induced by photochemical injury as described previously [18]. Mice 8 to 12 weeks of age were anesthetized by intraperitoneal injection with sodium pentobarbital and placed in the supine position on a dissecting microscope (Nikon SMZ-2T; Mager Scientific, Inc., Dexter, MI). A midline surgical incision was made to expose the right common carotid artery and a Doppler flow probe (model 0.5 VB; Transonic Systems, Ithaca, NY) is placed under the vessel. The probe was connected to a flowmeter (Transonic model T106) and was interpreted with a computerized data acquisition program (Windaq; DATAQ Instruments, Akron, OH). Rose Bengal [4,5,6,7-tetrachloro-3′, 6-dihydroxy-2,4,5,7-tetraio- dospiro (isobenzofuran-1(3H),9[9H] xanthan)-3:1 dipotassium salt; Fisher Scientific, Fair Lawn, NJ) at 50 mg/kg in 0.9% saline] was then injected into the tail vein in a 0.12-ml volume1. After injection into the tail vein, a green laser light (Melles Griot, Carlsbad, CA) at a wavelength of 540 nm was applied 6 cm from the carotid artery. Flow is monitored continuously from the onset of injury. The time to occlusion was determined only after the vessel remained closed with a cessation of blood flow for 20 min.

### Mouse inferior vena cava complete ligation venous thrombosis

Procedure was performed as previously described [19]. Briefly, mice aged 8–12 weeks were anesthetized with Isoflurane. Using sterile precautions, the abdominal cavity is opened with a ventral midline incision. All IVC back branches, from renal veins to the iliac bifurcation, are cauterized while side branches are ligated. The IVC is separated from the aorta, just inferior to the renal veins and completely ligated with 7–0 prolene. The laparotomy site is then closed. The mice are allowed to recover and observed post-operatively for 2 hours before returning to their original housing units. IVC thrombus were harvested and weighed at 48 hours.

### Coagulation assays

One stage prothrombin time (PT) and activate partial thromboplastin time (aPTT) were performed manually one plasma obtained from saline and TJ2 treated mice using the Helena Laboratories reagent and protocol (Cat. Nos. 5248 and 5387). Blood was drawn in 3.8% (0.129M) sodium citrate in the ratio of 1:9 for anticoagulant: whole blood. Whole blood was centrifuged immediately at 2000g for 15 minutes and platelet poor plasma stored in aliquots at -20 °C for batched analysis.

### Mouse bleeding time

Mice 8 to 12 weeks of age were anesthetized by intraperitoneal injection with sodium pentobarbital and placed in the supine position. Tail bleeding times were measured by transecting the tails of anesthetized mice (50 mg/kg sodium pentobarbital) 5 mm from the tip. The tail was placed in 10 ml of saline at 37°C, and the time to cessation of bleeding for 20 s was determined with a stopwatch.

### Mouse platelets for flow cytometry

Mouse whole blood was collected by IVC puncture and anticoagulated with buffered ACD (85 mM trisodium citrate, 83 mM dextrose, and 21 mM citric acid, pH 6.5) at a 1:6 ratio with whole blood. PRP was prepared by centrifugation of whole blood at 2300 g for 20 sec. After collecting the supernatant, the sample was centrifuged again at 2300 xg for 10 sec and the first supernatant was pooled with the second. Pooled PRP aliquots were then pelleted by centrifugation at 2200 g for 1 min per 200 μl of PRP and after removing the supernatant, the platelets were resuspended to desired platelet count with Hepes Tyrodes buffer, pH 7.4. Platelet flow cytometry was performed on washed platelets diluted to 0.5 X 10^8^/ml in HEPES-Tyrodes buffer. Platelets were stimulated with increasing concentrations of human α-thrombin (0.02-10nM) or ADP (1.25–20 μM). Thrombin-activated platelets were examined on flow cytometry for α_2b_β_3_ integrin activation with the JON/A antibody and P-selectin expression with the Wug.E9 antibody. ADP-stimulated washed platelets were examined for Alexa Fluor 488-labeled fibrinogen (Molecular Probes) binding on flow cytometry.

### RNA extraction and qPCR array (mouse NFkB signaling pathway)

RNA was extracted from mouse peritoneal macrophage by Roche High Pure RNA Isolation Kit (Roche). RNA quality was assessed by the Nanodrop ND-100 (Nanodrop Technologies, Inc., Wilmington DE). The CDNA was reverse transcribed by Iscript^™^ II. qPCR was performed with the TaqMan method (Roche Universal ProbeLibrary System) on a ViiA 7 Real-Time PCR System (Applied Biosystems). Relative expression was calculated using the ΔΔCt method with normalization to β-actin. All the experiments were repeated by at least 3 times by using independent samples. Mouse NFkB signaling pathway gene expression profiling was assessed with RT2 Profiler PCR Array (Qiagen, PAMM-025ZC-2) and β actin was used as control gene. The data were analyzed by GeneGlobe Data Analysis Center (Qiagen).

### Statistical analysis

All data, unless indicated are presented as the mean ± SEM. Two-tailed Student’s t test was used to compare the differences between two groups. One-way ANOVA and Bonferroni post test was used for multiple comparisons. Statistical significance was defined as P < 0.05.

## Results

### Assessing effect of TJ2 on cellular proliferative activity (MTS assay)

To assess whether TJ2 affects cellular proliferative activity, we treated RAW 264.7 cells with increasing concentrations of TJ2 for a maximum of 72 hours followed by MTS assay. All assays were performed in triplicate and control cells were maintained in growth medium only. We demonstrate that TJ2 concentrations of 5 mg/ml or lower did not significantly alter cell growth as compared to control medium (S2 Fig). Based on these results, TJ2 concentrations of 5mg/ml were used in the subsequent in vitro experiments.

### TJ2 attenuates cellular proinflammatory activation

**Fig 1:**
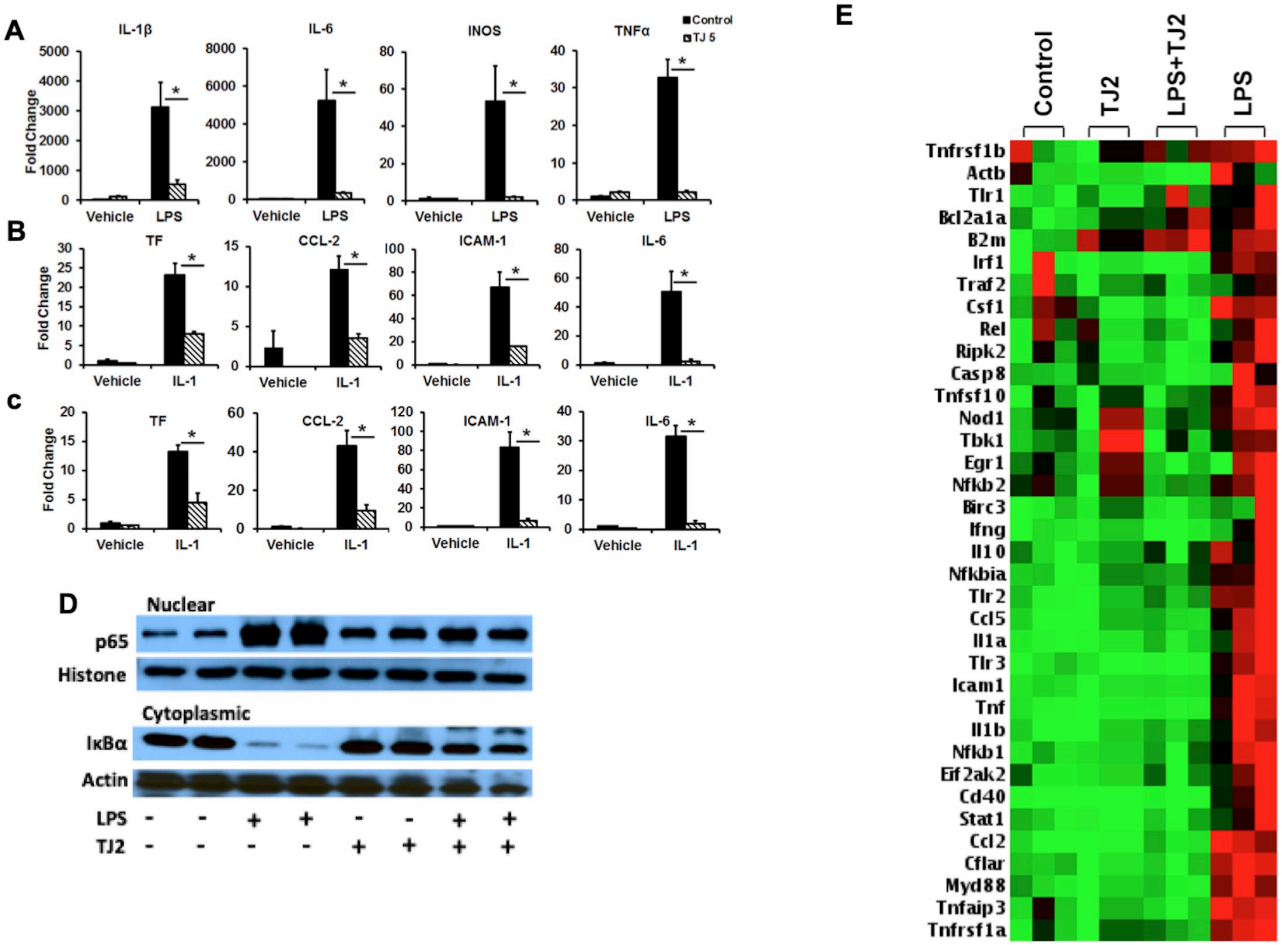
TJ2 regulates cellular inflammation. (A-C) RAW264.7 cells (A), HAEC (B) and HUVEC (C) treated with TJ2 for 24 hours followed by LPS for 6 hours and examined for inflammatory gene expression as indicated. (D) Nuclear and cytoplasmic extracts from RAW264.7 cells treated with TJ2 followed by LPS and immublotted for p65 and IκBα. (E) RT^2^ Profiler PCR Array (mouse NFkB signaling pathway) in RAW264.7 cells following vehicle versus TJ2 treatment followed by LPS treatment.

Since traditional Chinese medications are shown to affect the immune system, we sought to examine the effect of TJ2 on immune cells. Here, we used RAW264.7 cells to examine the effect of TJ2 on inflammation. These cells were pretreated with 5mg/ml TJ2 or vehicle for 24 h followed by 100 ng/ml LPS for 6h. The mRNA expression levels of IL-1β, IL-6, iNOS and TNFα were then examined. As shown in Fig 1A, LPS treatment is associated with a robust induction of IL-1β, IL-6, iNOS and TNFα mRNA. In contrast, induction is markedly attenuated in cells that received TJ2 treatment suggesting that TJ2 may have an anti-inflammatory effect.

We next sought to examine the anti-inflammatory effect of TJ2 on other cellular elements. Here, endothelial cell lines HAEC and HUVECs were pretreated with TJ2 or vehicle for 24 h followed by stimulation with IL-1 (100 ug/ml) for 6h. These samples were then examined for the expression of CCL-2, IL-6, ICAM-1 and tissue factor (TF). As shown in Fig 1B and 1C, IL-1 treatment was associated with a strong induction in the expression of CCL-2, IL-6, ICAM-1 and TF in vehicle treated cells, whereas, TJ2 pretreatment was associated with a significant decrease in the expression of these genes. Taken together, these results indicate that TJ2 strongly attenuates cellular activation in response to proinflammatory stimuli.

### TJ2 inhibits NF-kB activation by decreasing LPS-mediated IKBα degradation

Our studies show that TJ2 generates a robust anti-inflammatory effect. Given the central role of NF-κB in inflammation, we next sought to examine how TJ2 affects p65 expression upon cellular activation. Here, we treated RAW264.7 cells with LPS (100 ng/ml) for the indicated time points. Under quiescent conditions, p65 is bound to IκB and located in the cytosol. Upon cellular stimulation, IκB protein is rapidly phosphorylated by IκB kinase and degraded following which p65 is translocated to the nucleus [7]. To examine how TJ2 may affect this process, western blot analysis was performed using the nuclear and cytosolic fractions of RAW 264.7 cells. Fig 1D demonstrates that, as expected, nuclear p65 markedly increased following exposure to LPS alone, indicating LPS induced translocation of p65 from the cytosol to the nucleus. However, TJ2 pretreatment was associated with a significant decrease in p65 nuclear translocation following LPS treatment. Consistently, TJ2 pretreatment was also associated with a decrease in cytosolic IκB-α degradation following exposure to LPS as compared to controls. These findings indicate that TJ2 inhibits IκB-α degradation and consequently decreases nuclear translocation of p65.

### TJ2 exhibits broad anti-inflammatory effects in LPS-induced macrophages

To gain a comprehensive understanding of TJ2-mediated suppression of inflammation, we screened 84 genes related to the NF-KB pathway using a commercially available qPCR array (RT^2^ Profiler Array, Qiagen). RAW264.7 cells pretreated with either vehicle or TJ2 followed by LPS stimulation for 6 hours were examined. As shown in Fig 1E, our results demonstrate that a broad spectrum of NFκB target genes, including classic pro-inflammatory factors IL-1, TNF, CCL5, and CCL2, are induced by LPS treatment. However, the induction of these genes was significantly blocked by TJ2 treatment, indicating its broad anti-inflammatory effects. These data suggest that TJ2 significantly affects NF-KB activity and provides mechanistic basis for the ant-inflammatory effect noted with TJ2 treatment in cells.

### TJ2 alters arterial and venous thrombosis

**Fig 2:**
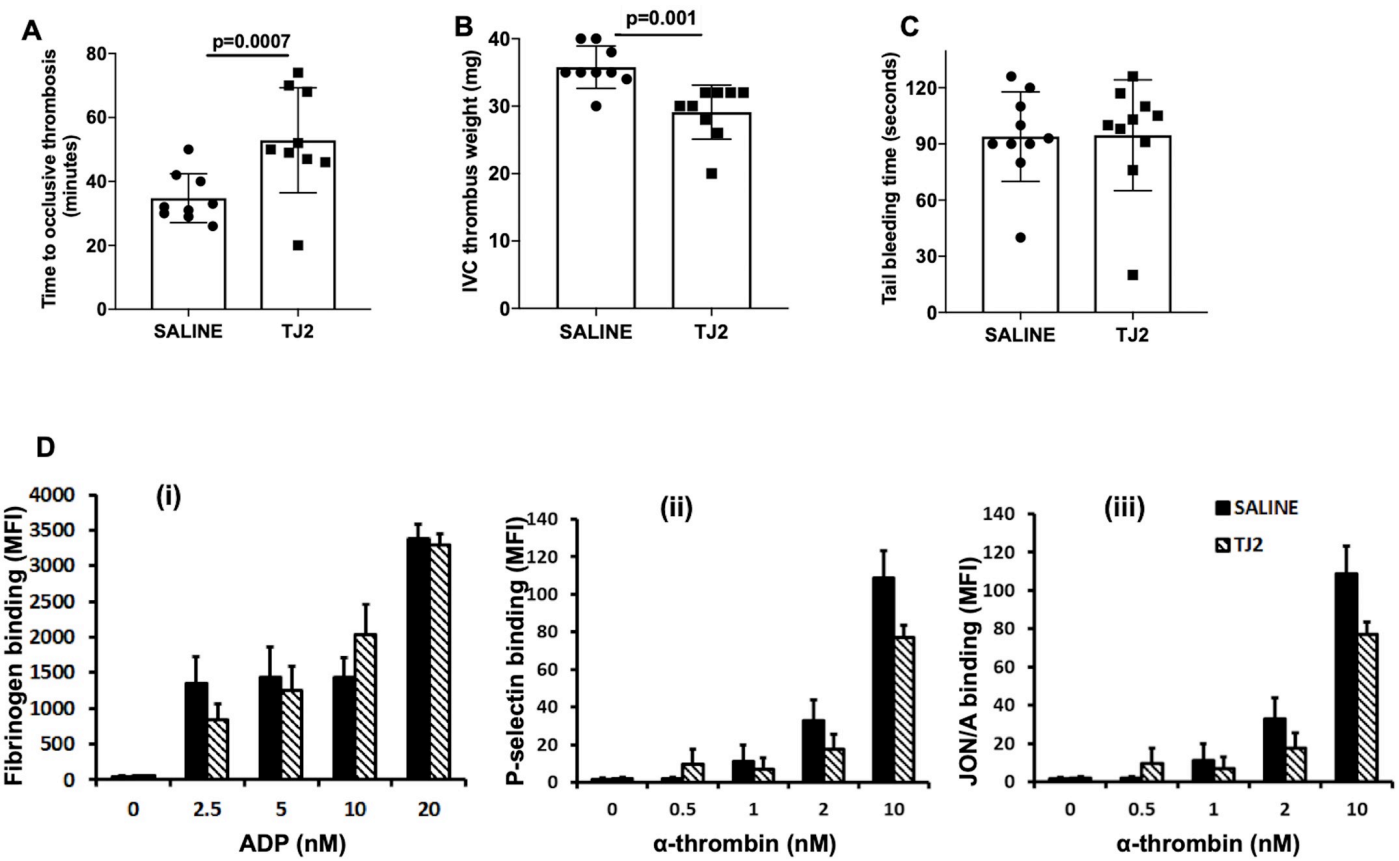
TJ2 ameliorates arterial and venous thrombosis. (A) Carotid artery thrombosis assay (Rose Bengal, photochemical injury model), (B) complete Inferior Vena Cava (IVC) ligation venous thrombosis assay, (C) Tail bleeding assay, and (D) Platelet activation assays in C57BL/6J mice treated with TJ2 (at 104mg/day) gavage for two weeks. The assay used flow cytometry to examine (i) JON/A binding to platelets stimulated with alpha-thrombin, (ii) P-selectin expression on platelets stimulated with alpha-thrombin, (iii) fibrinogen binding to platelets after stimulation of platelets with adenosine 5'-diphophate (ADP). The concentration-dependent results are the mean ± standard error of the mean of 5 individual experiments. All thrombosis studies were performed within 24 hours after the last dose of TJ2 was administered by gavage.

The endothelium and immune cells are implicated as major cellular determinants of coagulation. Emerging data demonstrates that an activated endothelium or immune cell can generate a proinflammatory and prothrombotic environment whereas maintaining an anti-inflammatory phenotype has an anti-thrombotic effect. Since our data indicates that TJ2 treatment has an anti-inflammatory effect, we postulated that TJ2 treatment would generate a thromboprotective effect. Mice underwent gavage daily (100 ul/day) with various concentrations of TJ2 reconstituted in saline (26, 52, 104 mg/mouse/day) for two weeks, Group 1 = 26 mg/mouse/day Group 2 = 52 mg/mouse/day Group 3 = 104 mg/mouse/day, vehicle group received equal volume of PBS). At all the doses used, no change was noted in white blood cell (WBC) counts, platelet counts, red blood cell (RBC) counts. Next, C57BL/6J mice treated with TJ2 at 104 mg/mouse/day were subjected to carotid artery thrombosis by using the photochemical injury model. Mice treated with TJ2 demonstrated a significantly prolonged time to occlusive carotid artery thrombosis compared with mice treated with saline (34±8 vs 50±12 minutes; P = 0.0008; n = 8 mice per group) (Fig 2A). We next sought to examine the effect of TJ2 on venous thrombosis. Here, we used a mouse model of complete inferior vena cava ligation in TJ2 treated (at 104mg/day for two weeks) versus saline treated mice. As shown in Fig 2B, mice treated with TJ2 demonstrated a significant decrease in the weight of the thrombus as compared to those in mice treated with saline (35.25±3vs 30.25±2 mg; P = 0.0018; n = 8 mice per group). No difference was noted in the coagulation assay (prothrombin time or PT and activated partial thromboplastin time or aPTT, S2 Fig), tail-bleeding time (Fig 2C) or in platelet activation assays (Fig 2D) performed on saline versus TJ2 treated mice (treated at 104mg/day of TJ2, as in mice treated for thrombosis assays).

Collectively, these results show that TJ2 treatment has an anti-thrombotic effect that is not associated with increased bleeding or alterations in platelet activation.

## Discussion

Inflammation is the initial innate immune response to a variety of pathological stimuli and tissue injuries and anti-inflammatory medications are the mainstay of treatment for immunological disorders that are associated with marked increase in inflammation. At the same time, accumulating evidence identifies an intimate link between inflammation and thrombosis and systemic inflammation is widely recognized as a potent risk for thrombosis [7, 14]. The importance of the inflammatory response in venous thrombosis has been shown in in vitro studies and in animal models. Similarly, inflammation is a significant risk factor for cardiovascular disease [20]. Circulating inflammatory cytokines cause vascular insult and atheromatous changes. Consequently, medications that decrease inflammation may need to be considered in cardiovascular disorders.

The anti-inflammatory function of TJ2 is recognized in traditional Chinese medicine. The active ingredients contained within the TJ2 granules, as noted with HPLC-MS, such as astragaloside, chlorogenic acid and ginsenosides are recognized to contain anti-inflammatory properties [12]. In the present study, we examine the effect of TJ2 on both inflammation as well as thrombosis. Our results in both endothelial and immune cells demonstrate that TJ2 generates a pronounced anti-inflammatory effect. This is evidenced by a significant attenuation in the LPS-induced production of inflammatory cytokines such as IL-1β, TNF-α, IL-1. Given that NF-κB is a major transcriptional regulator of inflammation [14], we posited that TJ2 may affect NF-κB signaling leading to the anti-inflammatory effects noted in our studies. Consistently, using a NF-κB signaling pathway qPCR array, we demonstrate that TJ2 pre-treatment affects a broad range of NF-κB pathway genes confirming our initial observations with TJ2. In the quiescent state, NF-κB is retained in the cytoplasm by IκB; however, upon exposure to a pro-inflammatory stimulus such as LPS, IκB is rapidly degraded allowing for translocation of NF-κB into the nucleus followed by initiation of transcription of pro-inflammatory genes [14, 21]. Thus, inhibition of IκB degradation prevents NF-κB nuclear translocation and pro-inflammatory cytokine expression. In alignment with these observations, we show that TJ2 pre-treatment inhibits IκB degradation and nuclear translocation of NF-κB identifying the mechanistic basis for the anti-inflammatory effect noted with TJ2 pre-treatment.

The intimate link between inflammation and thrombosis affects a wide variety of pro-inflammatory disorders that are associated with an increased risk for thrombosis [14]. TJ2 treatment is associated with an anti-inflammatory effect. In addition, we note decrease in TF expression in TJ2 treated endothelial cells. Previously studies highlight the importance of endothelial and hematopoietic tissue factor in thrombosis [22]. Thus, we sought to examine the effect of TJ2 on thrombosis. Here, we show that TJ2 prolonged thrombosis times in the carotid artery photochemical injury assay in normal mice and decreased the size of thrombus in complete IVC ligation mice. Although these results do not delineate the precise cellular elements that are operative in the generation of this anti-thrombotic effect, based on our in vitro studies, it is likely that TJ2 treatment is associated with decreased expression of pro-thrombotic factors such as TF in the vasculature or in the immune cells, thus demonstrating an anti-thrombotic effect. Our future studies will pursue these aspects of research. Presently used anticoagulant agents such as heparin, warfarin, and anti-platelet drugs such as aspirin and clopidogrel are associated with an increased risk of bleeding. It is important to note that the antithrombotic effect of TJ2 was not associated with any change in peripheral blood counts, coagulation assays, as well as platelet activation assays suggesting the potential for TJ2 as a safer alternative for management of thrombotic disorders. At Tongji hospital, TJ2 is currently being studied for use in combination with anticoagulants in patients with venous thrombotic events and coronary artery disease. Our studies identify a mechanistic basis for possible antithrombotic benefit with this drug and provide a basis upon which clinical studies may be proposed.

TJ2 is an herbal preparation consisting of a combination of various active ingredients (as listed above). It is not clear from our studies whether the antithrombotic effects are secondary to one particular ingredient or the result of a combination of different chemicals. Future studies using combination of organic and active components analysis are warranted from this point of view. Since TJ2 generates an antithrombotic effect without affecting coagulation or platelet activation assays, it would be interesting to pursue further research with this herbal preparation. Identifying one or more active ingredients with antithrombotic properties would then allow more focused studies with these products. Insights gleaned from these studies have the potential to change the current paradigms of management of thrombotic disorders. The understanding that inflammation is a major driver of thrombosis allows us to change our focus from anticoagulation or antiplatelet agents to anti-inflammatory agents in our management of thrombotic disorders.

## Supporting information

S1 Fig MTS assay to examine cytotoxic effect of TJ2. RAW264.7 cells were treated at indicated doses of TJ2 for 72 hours followed by MTS assay.
(TIFF)

S2 Fig TJ2 treatment is not associated with alterations in peripheral blood counts or coagulation assays. (A-C) Peripheral blood counts, WBC, RBC, and Platelet counts in C57BL/6J mice treated with TJ2 gavage at indicated doses for two weeks; (D-E) coagulation assays (Prothrombin time, PT and activated partial thromboplastin time, aPTT) in C57BL/6J mice treated with TJ2 gavage at 104mg/day for two weeks. All assays were done 24 hours after last dose.
(TIFF)

S3 Fig Platelet flow cytometry from mice treated with TJ2 at 104mg/day gavage daily for two weeks versus vehicle. Figures showing flow cytometry results to fibrinogen binding following ADP treatment and JON/A expression following thrombin activation of platelets.
(TIFF)

S1 Raw Images (PDF)

## References

[1] Khan S, Choi RJ, Shehzad O, Kim HP, Islam MN, Choi JS, et al Molecular mechanism of capillarisin-mediated inhibition of MyD88/TIRAP inflammatory signaling in in vitro and in vivo experimental models. Journal of ethnopharmacology. 2013;145(2):626–37. 10.1016/j.jep.2012.12.001 23237934

[2] Khan S, Shin EM, Choi RJ, Jung YH, Kim J, Tosun A, et al Suppression of LPS-induced inflammatory and NF-kappaB responses by anomalin in RAW 264.7 macrophages. Journal of cellular biochemistry. 2011;112(8):2179–88. 21480361 10.1002/jcb.23137

[3] Chao LK, Liao PC, Ho CL, Wang EI, Chuang CC, Chiu HW, et al Anti-inflammatory bioactivities of honokiol through inhibition of protein kinase C, mitogen-activated protein kinase, and the NF-kappaB pathway to reduce LPS-induced TNFalpha and NO expression. Journal of agricultural and food chemistry. 2010;58(6):3472–8. 10.1021/jf904207m 20192217

[4] Makarov SS. NF-kappaB as a therapeutic target in chronic inflammation: recent advances. Molecular medicine today. 2000;6(11):441–8. 10.1016/s1357-4310(00)01814-1 11074370

[5] Jou IM, Lin CF, Tsai KJ, Wei SJ. Macrophage-mediated inflammatory disorders. Mediators of inflammation. 2013;2013:316482 10.1155/2013/316482 23843681 PMC3697412

[6] Wadleigh DJ, Reddy ST, Kopp E, Ghosh S, Herschman HR. Transcriptional activation of the cyclooxygenase-2 gene in endotoxin-treated RAW 264.7 macrophages. The Journal of biological chemistry. 2000;275(9):6259–66. 10.1074/jbc.275.9.6259 10692422

[7] Esmon CT. Inflammation and thrombosis. Journal of thrombosis and haemostasis: JTH. 2003;1(7):1343–8. 12871267 10.1046/j.1538-7836.2003.00261.x

[8] Yau JW, Teoh H, Verma S. Endothelial cell control of thrombosis. BMC cardiovascular disorders. 2015;15:130 10.1186/s12872-015-0124-z 26481314 PMC4617895

[9] Nagareddy P, Smyth SS. Inflammation and thrombosis in cardiovascular disease. Current opinion in hematology. 2013;20(5):457–63. 10.1097/MOH.0b013e328364219d 23892572 PMC4086917

[10] Holmqvist ME, Neovius M, Eriksson J, Mantel A, Wallberg-Jonsson S, Jacobsson LT, et al Risk of venous thromboembolism in patients with rheumatoid arthritis and association with disease duration and hospitalization. Jama. 2012;308(13):1350–6. 23032551 10.1001/2012.jama.11741

[11] Ridker PM, Everett BM, Thuren T, MacFadyen JG, Chang WH, Ballantyne C, et al Antiinflammatory Therapy with Canakinumab for Atherosclerotic Disease. The New England journal of medicine. 2017;377(12):1119–31. 10.1056/NEJMoa1707914 28845751

[12] Fang Yu W L-L, Zhu Jin-hui, Zhu De-qiu. Determination of astragaloside IV, chlorogenic acid, ginsenosides Rg1, Rb1, and notoginsenoside R1 in Tongji No. 2 granules by HPLC-MS/MS. Drug Evaluation Research. 2015;38(4):394–7.

[13] Yang X, Xiong X, Wang H, Wang J. Protective effects of panax notoginseng saponins on cardiovascular diseases: a comprehensive overview of experimental studies. Evidence-based complementary and alternative medicine: eCAM. 2014;2014:204840. 25152758 10.1155/2014/204840 PMC4131460

[14] Achyut BR, Arbab AS. Myeloid cell signatures in tumor microenvironment predicts therapeutic response in cancer. OncoTargets and therapy. 2016;9:1047–55. 10.2147/OTT.S102907 27042097 PMC4780185

[15] Qiu LH, Zhang BQ, Lian MJ, Xie XJ, Chen P. Vascular protective effects of Astragalus membranaceus and its main constituents in rats with chronic hyperhomocysteinemia. Experimental and therapeutic medicine. 2017;14(3):2401–7. 10.3892/etm.2017.4739 28962174 PMC5609140

[16] Nikzad-Langerodi R, Ortmann S, Pferschy-Wenzig EM, Bochkov V, Zhao YM, Miao JH, et al Assessment of anti-inflammatory properties of extracts from Honeysuckle (Lonicera sp. L., Caprifoliaceae) by ATR-FTIR spectroscopy. Talanta. 2017;175:264–72. 28841989 10.1016/j.talanta.2017.07.045

[17] Xiong Y. A new method to calculate drug dosage from human to experimental animals. Acta Academiae Medicinae Jiangxi. 1997;04:41.

[18] Nayak L, Shi H, Atkins GB, Lin Z, Schmaier AH, Jain MK. The thromboprotective effect of bortezomib is dependent on the transcription factor Kruppel-like factor 2 (KLF2). Blood. 2014;123(24):3828–31. 10.1182/blood-2014-01-547448 24771858 PMC4055929

[19] Wrobleski SK, Farris DM, Diaz JA, Myers DD Jr., Wakefield TW. Mouse complete stasis model of inferior vena cava thrombosis. Journal of visualized experiments: JoVE. 2011(52). 10.3791/2738 21712794 PMC3346045

[20] Berg AH, Scherer PE. Adipose tissue, inflammation, and cardiovascular disease. Circulation research. 2005;96(9):939–49. 10.1161/01.RES.0000163635.62927.34 15890981

[21] Takeda K, Kaisho T, Akira S. Toll-like receptors. Annual review of immunology. 2003;21:335–76. 10.1146/annurev.immunol.21.120601.141126 12524386

[22] Pawlinski R, Wang JG, Owens AP 3rd, Williams J, Antoniak S, Tencati M, et al Hematopoietic and nonhematopoietic cell tissue factor activates the coagulation cascade in endotoxemic mice. Blood. 2010;116(5):806–14. 10.1182/blood-2009-12-259267 20410508 PMC2918334

