# Correction: The thromboprotective effect of traditional Chinese medicine Tongji 2 granules is dependent on anti-inflammatory activity by suppression of NF-κB pathways

**DOI:** 10.1371/journal.pone.0250139

**Published:** 2021-04-08

**Authors:** Lin Zhou, Stephanie Lapping, Xudong Liao, Yuan Lu, Guangjin Zhou, Keiichiro Matoba, Neelakantan T Vasudevan, Lemin Wang, Lalitha Nayak

The seventh author’s name is spelled incorrectly. The correct name is: Neelakantan T Vasudevan.
