# Supplementary figures and images for: The thromboprotective effect of traditional Chinese medicine Tongji 2 granules is dependent on anti-inflammatory activity by suppression of NF-κB pathways
Source: PLoS One. 2020 Nov 12;15(11):e0241607. doi: 10.1371/journal.pone.0241607 (PMC7660536; doi:10.1371/journal.pone.0241607)

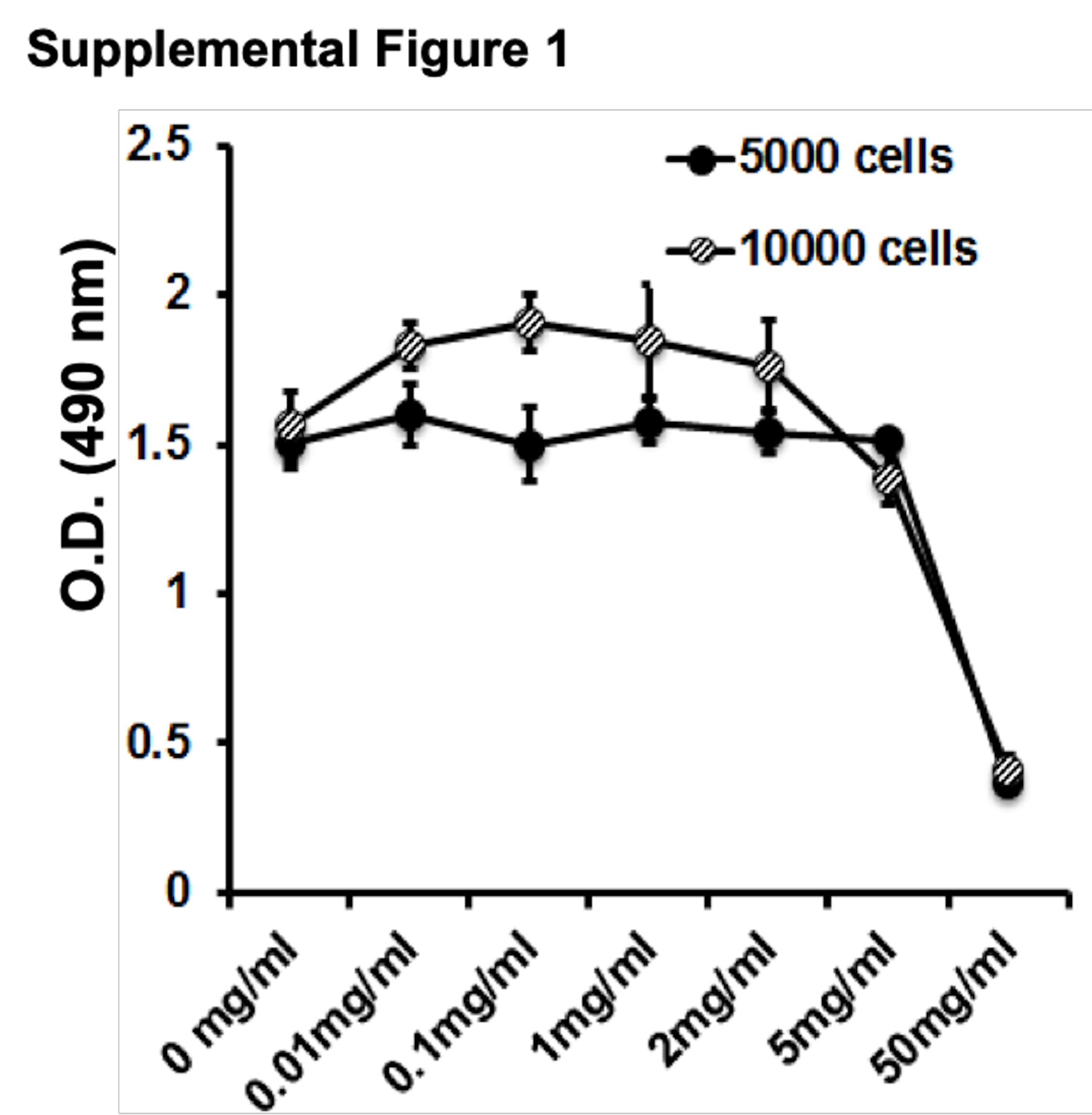

Supplement: S1 Fig — RAW264.7 cells were treated at indicated doses of TJ2 for 72 hours followed by MTS assay. (TIFF) [file pone.0241607.s001.tiff]

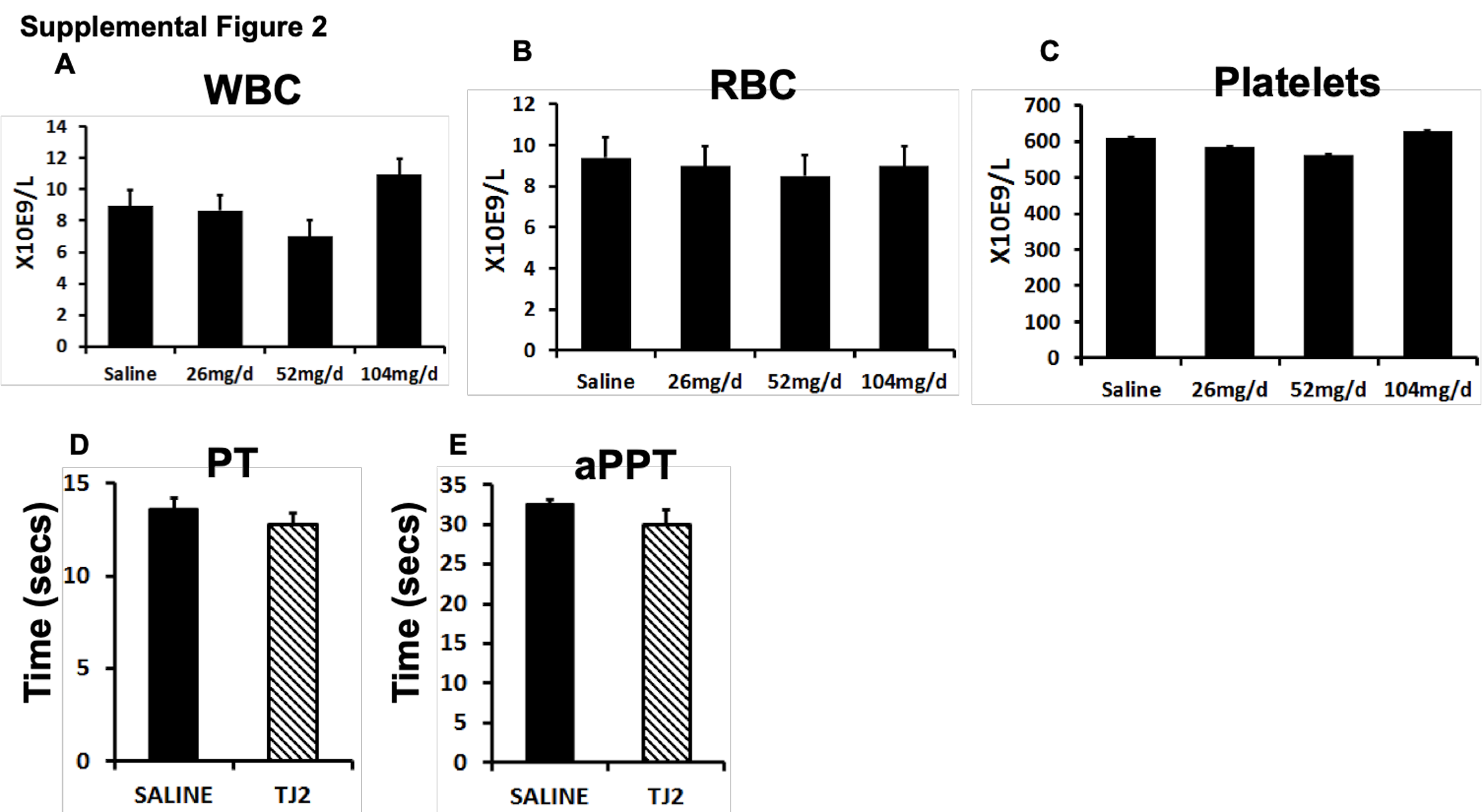

Supplement: S2 Fig — (A-C) Peripheral blood counts, WBC, RBC, and Platelet counts in C57BL/6J mice treated with TJ2 gavage at indicated doses for two weeks; (D-E) coagulation assays (Prothrombin time, PT and activated partial thromboplastin time, aPTT) in C57BL/6J mice treated with TJ2 gavage at 104mg/day for two weeks. All assays were done 24 hours after last dose. (TIFF) [file pone.0241607.s002.tiff]

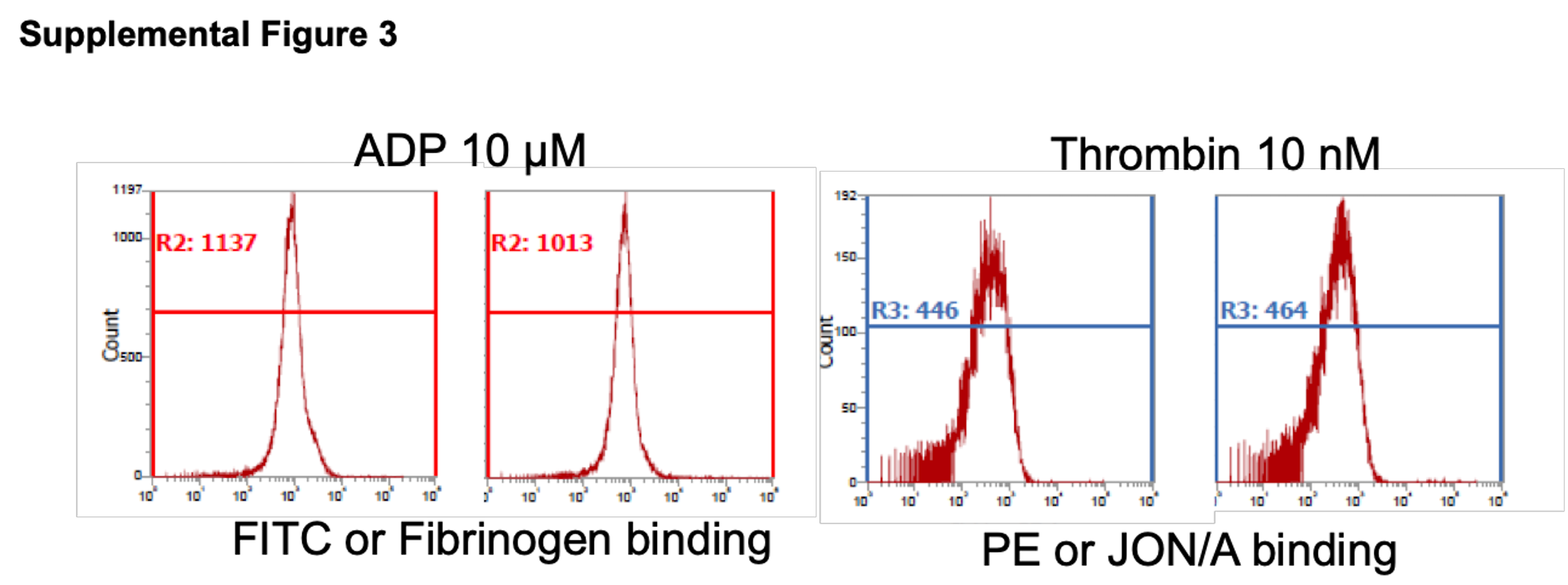

Supplement: S3 Fig — Figures showing flow cytometry results to fibrinogen binding following ADP treatment and JON/A expression following thrombin activation of platelets. (TIFF) [file pone.0241607.s003.tiff]
