# Supplementary material for: The thromboprotective effect of traditional Chinese medicine Tongji 2 granules is dependent on anti-inflammatory activity by suppression of NF-κB pathways
Source: PLoS One. 2020 Nov 12;15(11):e0241607. doi: 10.1371/journal.pone.0241607 (PMC7660536; doi:10.1371/journal.pone.0241607)
Supplement: S1 Raw Images — (PDF) [file pone.0241607.s004.pdf]

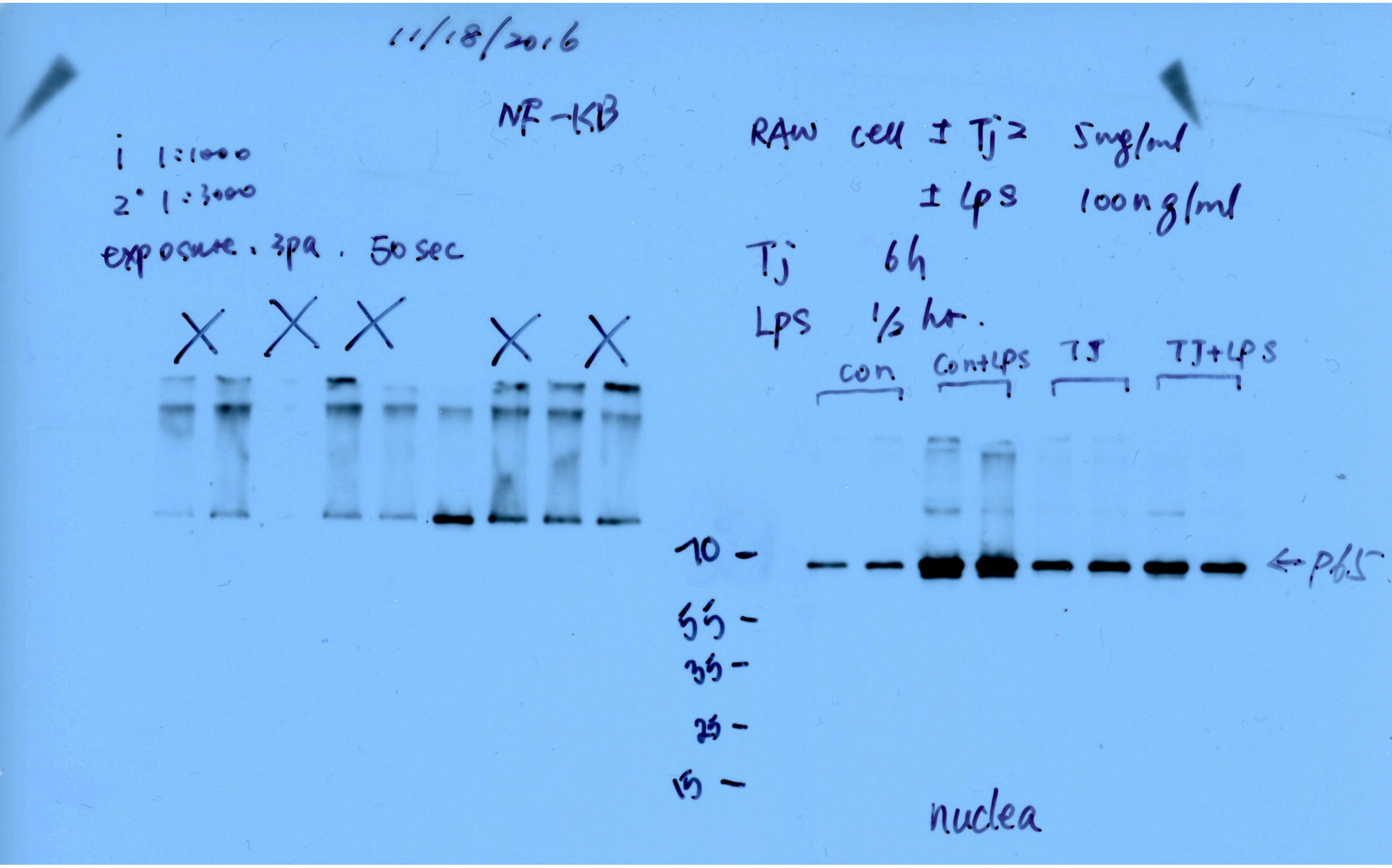
11/18/2016
NF-KB
1° 1:1000
2° 1:3000
exposure . 3pa . 50 sec
RAW cell ± Tj = 5ug/ml
± Lps 100ng/ml
Tj 6h
Lps 1/2 hr.
con
Con+LPS
TJ
TJ+LPS
70 -
55 -
35 -
25 -
15 -
← p65
nuclea

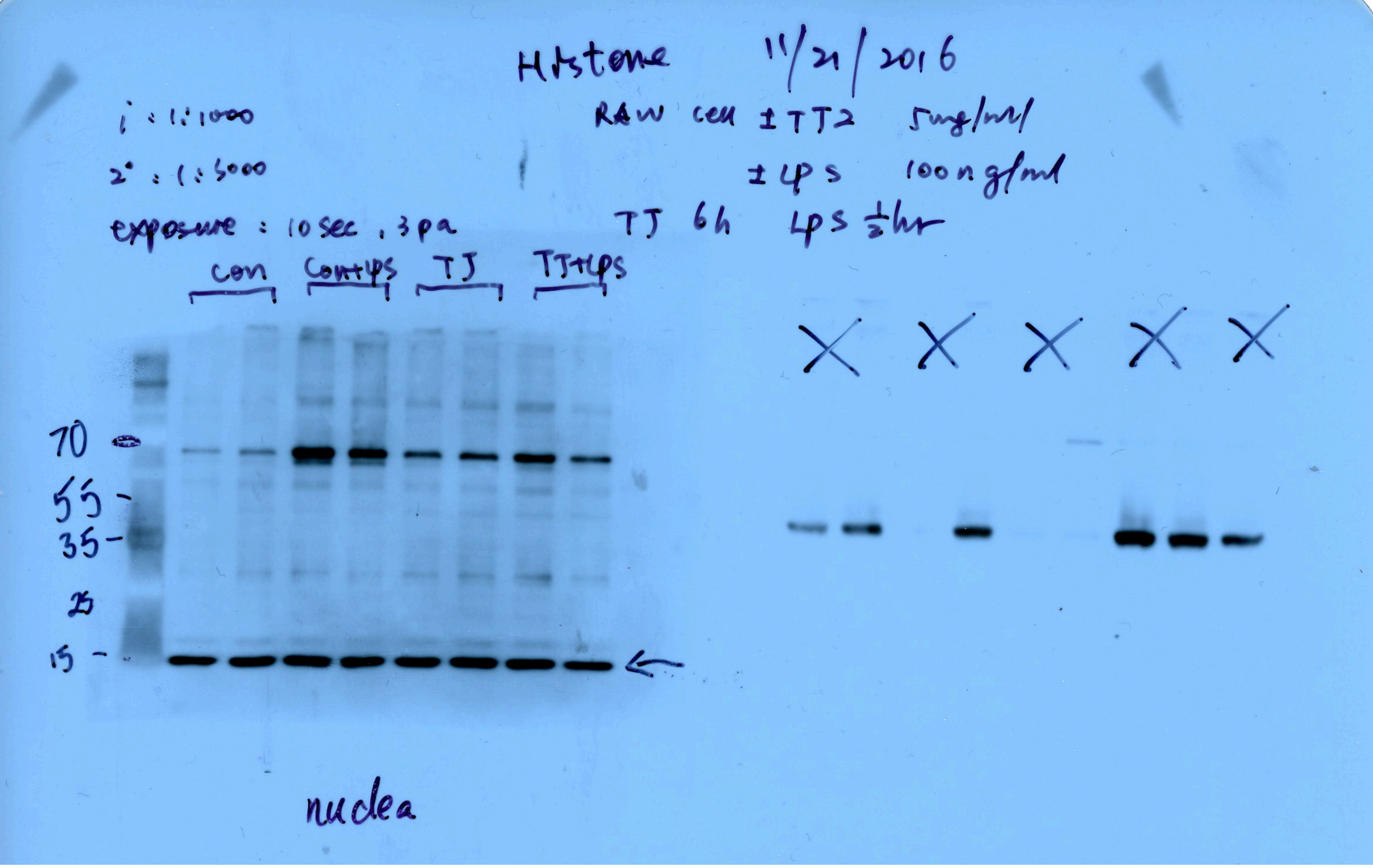
Histone 11/21/2016
1°: 1:1000
2°: 1:5000
exposure: 10 sec, 3pa
RAW cell ± TJ2 5ug/ml
± LPS 100ng/ml
TJ 6h LPS ½hr
Con
Con+LPS
TJ
TJ+LPS
70
55
35
25
15
nuclea

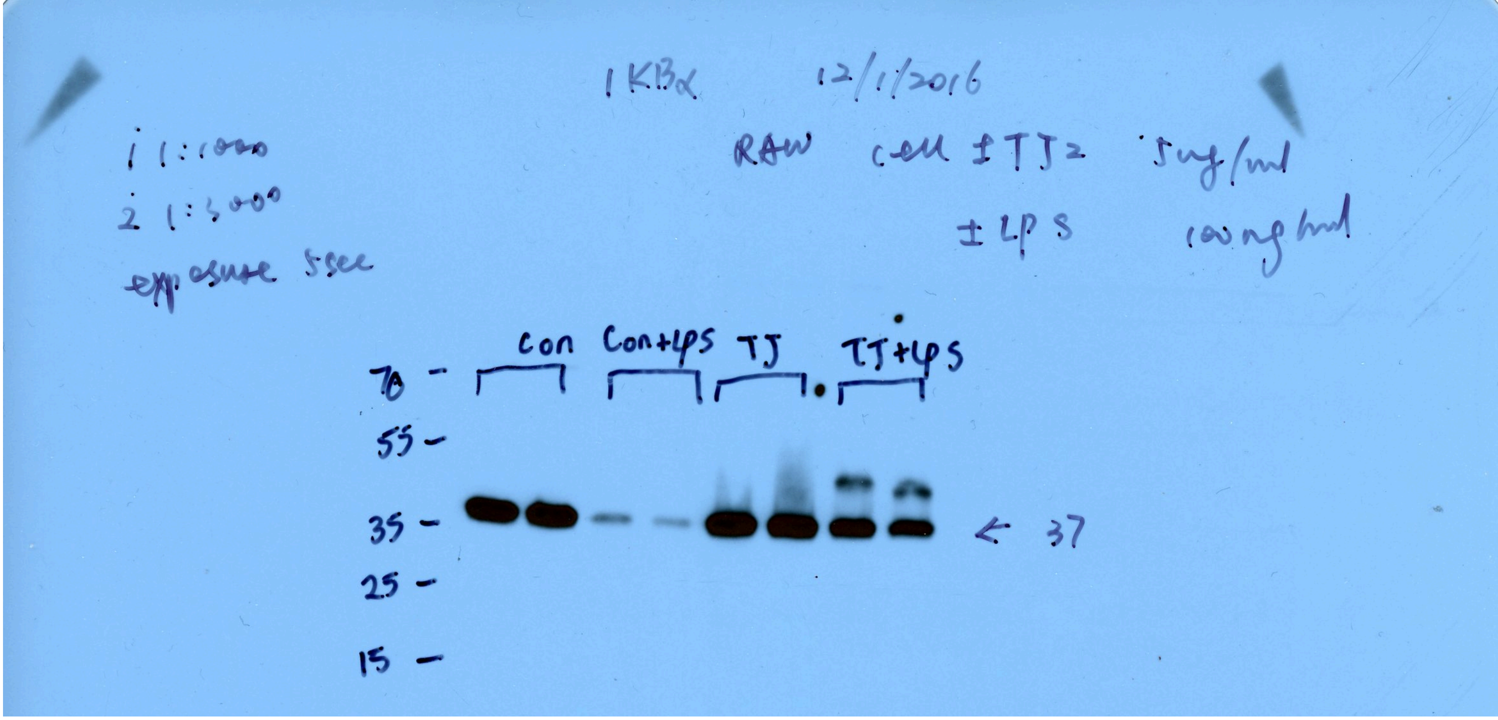
IKBα 12/1/2016
1 1:1000
2 1:5000
exposure 5sec
RAW cell ± TJ2 5ug/ml
± LPS 100ng/ml
Con
Con+LPS
TJ
TJ+LPS
70
55
35
25
15
← 37

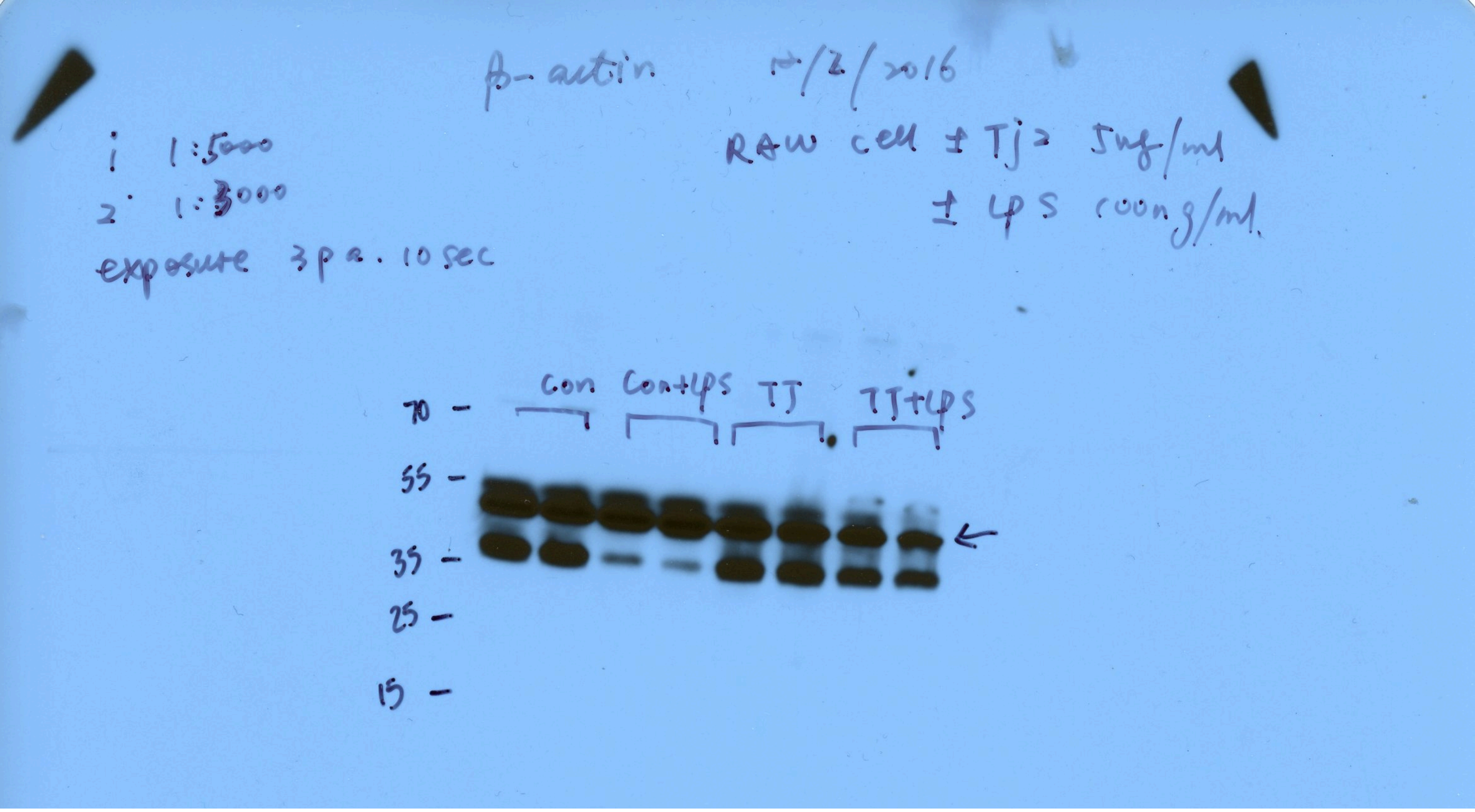
β-actin
10/2/2016
1: 1:5000
2: 1:3000
exposure 3pa. 10sec
RAW cell ± TJ2 5ug/ml
± LPS 100ng/ml
70
55
35
25
15
con
Con+LPS
TJ
TJ+LPS
